# Mining Microbial Niches: Sources of Bacteria for Enhancing Plant Growth and Resilience to pH, Salinity, Drought and *Phytophthora infestans*

**DOI:** 10.1111/1758-2229.70217

**Published:** 2025-10-18

**Authors:** Chiara Antonelli, Michele Narduzzi, Maurizio Ruzzi, Antonino Testa, Anna Maria Vettraino

**Affiliations:** ^1^ Department for Innovation in Biological, Agro‐Food and Forest Systems (DIBAF) University of Tuscia Viterbo Italy; ^2^ Department of Environmental, Biological and Pharmaceutical Sciences and Technologies University of Campania ‘Luigi Vanvitelli’ Caserta Italy

**Keywords:** abiotic stress, biocontrol, mycorrhizal roots, peperino fountain, *Phytophthora infestans*, plant growth‐promotion, straw digestate

## Abstract

Enhancing plant resilience is crucial for sustainable agriculture and ecosystem health in an era of climate change and the global spread of plant pathogens. The strategic exploitation of diverse ecological niches to isolate microorganisms that promote plant growth, suppress pathogens and tolerate abiotic stress conditions is paramount. Here, 21 bacterial strains were isolated from three distinct sources: digestate produced from straw feedstock, *Quercus ilex* roots mycorrhised with Tuber aestivum, and a peperino stone fountain. These isolates were assigned to eight genera, *Bacillus*, *Pseudomonas*, *Stenotrophomonas*, *Burkholderia*, *Acinetobacter*, *Aeromonas*, *Exiguobacterium* and *Sphingobacterium*, all linked to plant growth promotion, stress mitigation and pathogen control. Tests for plant‐growth‐promoting and biocontrol activities included indole acetic acid, siderophores, hydrogen cyanide, cellulase and chitinase and phosphate/potassium solubilisation. Efficacy against *Phytophthora infestans* and tolerance to salinity, pH and drought were also assessed. Functional patterns varied by isolation source, suggesting niche‐specific adaptation. Although all isolates displayed metabolic versatility, peperino stone strains exhibited higher tolerance to acidity and drought, while isolates from straw digestate and mycorrhizal roots tolerated alkalinity. Most isolates are promising for *P. infestans* control. These findings highlight the importance of targeted microbial sourcing in developing effective biofertilisers and biopesticides, offering sustainable solutions to agricultural and environmental challenges.

## Graphical abstract

In this study, 21 bacterial strains were isolated from diverse ecological niches, including straw digestate, mycorrhizal roots and a stone fountain. These strains, spanning eight genera, were evaluated for plant growth promotion, biocontrol against *Phytophthora infestans* , and tolerance to abiotic stresses such as salinity, pH and drought. Functional traits varied by source, with peperino isolates showing high drought and acidity tolerance, and several strains emerging as promising candidates for sustainable crop protection.

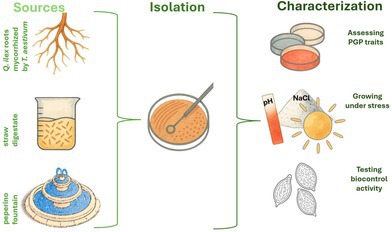

## 1 Introduction

Plants face a broad spectrum of challenges from abiotic and biotic factors, including extreme temperature fluctuations, increased soil salinity, drought and pest and pathogen diseases. These factors can significantly affect plant growth patterns and lead to a decline in crop yield quality. For example, production levels of essential crops such as maize and wheat experienced reductions of 3.8% and 5.5%, respectively, due to temperature trends observed from 1980 to 2008 (Dong et al. 2017; Lobell et al. 2011; Senapati et al. 2021). This situation is expected to worsen as the ongoing climate change. By 2050, average global temperatures are projected to rise by approximately 1.5°C, adversely affecting arable land through processes such as salinization (Collins et al. 2013; Jamil et al. 2011). Abiotic stresses often have synergistic effects (Nadeem et al. 2023; Ploschuk et al. 2025). For instance, droughts may coincide with heat waves, or salinity may coexist with nutrient deficiencies, complicating plant adaptation and survival (Abobatta 2020; Mittler 2006; Prasad et al. 2015). Climate change could also contribute to the spread of new pathogens introduced through plant trade, creating favourable conditions for their establishment and proliferation, posing significant threats to both forest, urban and agricultural ecosystems (Antonelli et al. 2022, 2024; Bebber et al. 2013; Franić et al. 2023; Jung et al. 2016; Vettraino et al. 2025). Historically, plant diseases have had profound societal, environmental and economic impacts. A notable example is the Great Irish Famine, which was caused by a highly destructive oomycete pathogen ( *Phytophthora infestans* (Mont.) de Bary) and resulted in approximately 700,000 deaths in Ireland, prompting a similar number of individuals to emigrate (Austin Bourke 1964). This pathogen spreads rapidly in cool, humid conditions, forming sporangia that release motile zoospores capable of infecting plant tissues. *Phytophthora infestans* exhibits high genetic diversity, allowing it to evolve rapidly and overcome resistant plant varieties and fungicides. Thus, it remains one of the most challenging plant pathogens to manage. The impact of *P. infestans* is still significant in modern agriculture, causing billions of dollars in losses annually, about USD 5 billion (Bahramisharif and Rose 2019; Nowicki et al. 2012), due to reduced yields and increased costs for fungicide applications. Efforts to control *P. infestans* have been ongoing for more than a century. Despite the wide range of strategies employed, the pathogen continues to cause devastating agricultural losses on a global scale (Haverkort et al. 2009). Although chemical fungicides are still commonly used against *P. infestans* , they pose significant environmental and economic challenges. Consequently, the use of Plant Growth‐Promoting Rhizobacteria (PGPR) is gaining importance as a sustainable alternative.

The European Union enhances plant resilience to abiotic and biotic stress through a comprehensive approach that integrates research initiatives, policy frameworks and funding programs (Eschen et al. 2015; Jeger et al. 2017; Nahrung et al. 2023; Regulation (EU) 2016/2031 2025; Regulation (EU) 1143/2014 2019; Vettraino et al. 2018).

The use of PGPR and Biological Control Agents (BCA) aligns with the EU Green Deal and Farm to Fork Strategy, which emphasises reducing chemical inputs and promoting environmentally friendly agricultural practices. Particularly, PGPR promote plant development and productivity by facilitating nutrient uptake and synthesising growth‐promoting compounds. They also play a crucial role as BCA, for instance, by producing siderophores and antimicrobial antibiotics and inducing systemic resistance in plants (Abou Jaoudé et al. 2024; Aeron et al. 2020; El‐Saadony et al. 2022; Haas and Keel 2003; Jiao et al. 2021). Therefore, identifying and utilising these microbial resources holds significant potential for supporting sustainable agricultural practices and ensuring food security and forest and urban greening health in response to growing environmental challenges. The deliberate selection of niches, including stress‐prone ecosystems, enhances the likelihood of isolating microbes with functional attributes such as tolerance to salinity, drought, mineral solubilisation, enzyme production and antagonism against phytopathogens. This study aimed to characterise various bacterial strains obtained from diverse sources, including the liquid fraction of digestate produced from straw feedstock, the roots of *Quercus ilex* colonised by ectomycorrhizal fungus *Tuber aestivum*, and a fountain made of peperino stone. Special emphasis was placed on pointing out which of the analysed sources were most likely to harbour microorganisms as promising candidates for efficient plant growth promotion and biocontrol applications against *P. infestans* , even under stress conditions.

## 2 Materials & Methods

### 2.1 Isolation of Bacteria for In Vitro Test

Bacteria were isolated from samples randomly collected from: (A) Liquid fraction of straw digestate (LDS); (B) Roots of *Quercus ilex* colonised by ectomycorrhizal fungus *Tuber aestivum* (QiR); (C) peperino fountain (PF). Serial dilutions were performed by taking 1 mL of LDS, 1 g of *Q. ilex* roots, and 6 samples from 2 square areas (10 cm^2^ of the fountain collected with sterile cotton swabs). Diluted samples were spread onto Luria Bertani medium (25 g/L, 20 g/L agar). Plates were incubated at 28°C for 48 h. Based on macroscopic features, bacterial colonies were transferred into new LB plates to obtain pure cultures. After a visual examination, bacterial isolates were grouped according to their morphology, and one strain was chosen to represent each cluster. Isolates were stored at −80°C in 30% (v/v) glycerol in the AMV culture collection (DIBAF, University of Tuscia, Italy).

### 2.2 Molecular Identification of Bacterial Isolates

Pure colonies were selected from the plates using sterile pipette tips, suspended in 100 μL of sterile water, and subjected to heat shock at 95°C for 10 min. The 16S rRNA region was amplified with 27F (5′‐AGAGTTTGATCCTGGCTCAG‐3′) and 1495R (5′‐CTACGGCTACCTTGTTACGA‐3′) primers, following the methodology of Sharma et al. (2008). DNA quality was assessed by agarose gel electrophoresis. The amplified products were purified using the NucleoSpin kit (Macherey‐Nagel GmbH & Co., Duren, Germany). Sequencing reactions were conducted by Eurofins Genomics (Cologne, Germany), and the forward and reverse sequences were assembled and edited using BioEdit Sequence Alignment Editor v7.0.5.3 software (Ibis Bioscience, CA, USA). Finally, phylogenetic trees were constructed using the Neighbor‐Joining method with the Kimura 2‐parameter, implemented in MEGA 11 software (Kimura 1980; Tamura et al. 2021). Bootstrap analysis was based on 1000 replications.

### 2.3 In Vitro Assessment of PGP, Metabolite‐Related, and Stress Resitance Properties of Bacterial Isolates and their Antagonistic Activity Against *Phytophthora infestans*

The following described screening experiments were performed in triplicate, and all the assays were repeated at least twice. This experimental design was consistently applied to all the tests described in this section.

#### 2.3.1 Qualitative Estimation of Phosphate and Potassium Solubilisation

National Botanical Research Institute's phosphate agar medium (NBRIP; 10.0 g glucose, 5.0 g Ca_3_(PO_4_)_2_, 5.0 g MgCl_2_.6H_2_O, 0.25 g MgSO_4_.7H2O, 0.2 g KCl, 0.1 g (NH_4_)_2_SO_4_, 20.0 g agar/L) and Aleksandrov medium (5.0 g glucose, 0.5 g MgSO_4_.7H_2_O, 0.1 g CaCO_3_, 0.006 g FeCl_3_, 2.0 g Ca_3_(PO_4_)_2_, 3.0 g potassium aluminium silicate (MICA), 20.0 g agar/L) were used to screen the bacteria for phosphate and potassium solubilisation (Etesami et al. 2017; Nautiyal 1999), respectively. After 5 days, halo zones surrounding the colony were measured.

#### 2.3.2 Quantitative Estimation of Indole‐3‐Acetic Acid (IAA) Production

The colorimetric method described by Tiryaki and Gülmez (2021) determined the production of indole acetic acid with slight modifications. Cultures were inoculated into 5 mL of LB medium supplemented with 0.1% L‐tryptophan and incubated at 30°C with constant shaking at 150 rpm. Samples were collected after 48 and 120 h of incubation and centrifuged at 11,000 rpm for 10 min. The supernatant from each isolate was mixed with Salkowski's reagent (150 mL of concentrated H_2_SO_4_, 250 mL of distilled water, 7.5 mL of 0.5 M FeCl_3_·6H_2_O) at a 1:2 (v/v) ratio. After 20 min at room temperature in the dark, the absorbance was measured at 530 nm using a UV–VIS spectrophotometer (Onda, UV‐30 SCAN). A standard curve was used to calculate the concentration of IAA, expressed as μg/mL.

#### 2.3.3 Siderophores Production

The Chrome Azurol Sulfonate (CAS) assay was used to evaluate the bacteria's ability to produce siderophores (Pranaw et al. 2020). Briefly, the CAS staining solution was prepared from three different solutions: (a) 60 mg Chromazurol S dissolved in 50 mL of distilled water; (b) 1 mM FeCl_3_.6H_2_O in 10 mL of 10 mM HCl; (c) 72.9 mg CTAB (Cetyltrimethylammonium bromide) in 40 mL of distilled water. Solutions were mixed and autoclaved at 121°C for 20 min. One hundred millilitre of the sterile CAS staining solution was added to 300 mL of sterile nutrient agar. The bacteria were then inoculated on the CAS agar medium at 28°C. After 2 days, the presence of a yellow halo around the colony indicated the production of siderophores.

#### 2.3.4 Chitinolytic Activity

Chitinolytic strains were screened in CHDA medium consisting of 0.65 g/L NaHPO_4_, 1.5 g/L KH_2_PO_4_, 0.5 g/L NH_4_Cl, 0.12 g/L MgSO_4_, 0.005 g/L CaCl_2_, 0.25 g/L NaCl and 20.0 g/L agar at pH 6.5, supplemented with 10% (w/v) colloidal chitin (Drewnowska et al. 2020). Colloidal chitin was prepared as described by Kuddus and Ahmad (2013). Briefly, 5 μL of each strain was inoculated onto CHDA medium plates and incubated at 30°C. After 10 days, the chitinolytic activities of the isolates were assessed by visualising the clear zone surrounding the colonies.

#### 2.3.5 Cellulolytic Activity

Bacterial cultures were screened for cellulase enzyme production on Congo red agar media (0.5 g KH_2_PO_4_, 0.25 g MgSO_4_, 2 g cellulose, 15 g agar, 0.2 g Congo red, 2 g gelatin, distilled water 1 L, pH 6.8–7.2) (Gupta et al. 2012). The plates were incubated at 30°C for 10 days. Bacteria that showed discoloration surrounding the colony were considered positive.

#### 2.3.6 Hydrogen Cyanide (HCN) Production

For estimating HCN production, we used the method proposed by Fiodor et al. (2023). Briefly, bacteria were streaked on King's B agar plates supplemented with 4.4 g/L glycine. A sheet of filter paper soaked in a 0.5% picric acid solution mixed with 2% sodium carbonate was placed on the surface of the medium. The plates were incubated at 30°C for 5 days. Positive bacteria (+) changed the colour of the filter paper from yellow to orange‐brown, while no colour change indicated a negative test (−).

#### 2.3.7 Abiotic Stress Tolerance

All tests were performed according to Fiodor et al. (2023).

##### 2.3.7.1 Salinity Tolerance

Bacteria were inoculated on LB agar with different concentrations of NaCl (5%, 7.5%, 10% and 15%) to evaluate salinity tolerance. Plates were incubated at 30°C for 5 days and observed for growth.

##### 2.3.7.2 pH Tolerance

Bacteria were tested for their ability to grow at different pH levels using LB agar medium with varying pH values (4, 5, 6, 8, 10). The pH of the LB media was adjusted to the desired levels using NaOH and HCl. Bacterial growth was visually assessed after 5 days at 30°C.

##### 2.3.7.3 Drought Stress

Bacteria were inoculated in LB broth with different concentrations of PEG 8000 (15.8%, 23.9% and 30.7%). Equation (1) was used to calculate the required PEG concentrations for generating different water potentials (Ψ) at given temperatures (*T*):

(1) $$\mathrm{PEG}=\left(4-\left(\;5.16\;\Psi T-56\text{0Ψ}+16\right)0.5\right)/\left(2.58T-280\right)$$

The inoculated flasks were incubated at 30°C for 5 days while shaking at 150 rpm. Sterile LB medium served as a control. Growth was measured using a Heales MB‐580 microplate reader at a wavelength of 600 nm.

#### 2.3.8 Seed Germination Test

Tomato “Roma VF” seeds were surface‐sterilised using 1% sodium hypochlorite for 2 min followed by 70% ethanol solution for 1 min (Pandey and Gupta 2020). The seeds were then shaken in an LB bacterial suspension (10^8^ CFU/mL) at 150 rpm for 2 h, while the control seeds were soaked in sterile LB broth medium. The germination test involved placing 15 seeds on sterile Whatman filter paper, which was moistened with 5 mL of either bacterial suspension or sterile medium for the control, and incubated for 7 days at 25°C in the growth chamber. At the end of the experiment, the germination index (GI) was estimated using the following formula:

(2) $$\mathrm{GI}=\left(\text{Number of germinated seeds}/\text{Total number of seeds}\right)\times 100$$

#### 2.3.9 Antagonistic Activity Against *Phytophthora infestans*

Each bacterial strain was streaked in the middle of a pea agar (120 g/L peas, 15 g/L agar) Petri plate and incubated at 30°C. The day after, two plugs (diameter 0.5 cm) of *P. infestans* AMV23 were placed at 0.5 cm from the opposite edges of the plate. Control plates were inoculated with *P. infestans* in the absence of bacteria. All plates were incubated at 20°C for 7 days. Percent inhibition was calculated using the following formula:

(3) $$\%\text{inhibition}=\left[1-\left(\text{Treatment growth}/\text{Control growth}\right)\right]\times 100$$

### 2.4 Data Analysis

The ability of the bacterial isolates to solubilise phosphate (PSI) and potassium (KSI), exhibit chitinolytic (CHI) and cellulolytic activities (CI) and produce siderophores (SPI) was qualitatively evaluated. The colony diameter and halo zone width were used to calculate the indices according to the following formula:

(4) $$\begin{array}{c} \mathrm{PSI}/\mathrm{KSI}/\mathrm{CHI}/\mathrm{CI}/\mathrm{SPI}\\ =\left(\text{diameter of the clearing zone}+\text{colony diameter}\right)/\text{colony diameter} \end{array}$$

Data shown in tables are mean values of two independent experiments with three repetitions. Before conducting ANOVA, the normality of distribution and homogeneity of variance were checked with Shapiro and Bartlett tests, respectively. Differences among treatments were analysed by one‐way ANOVA followed by Tukey's HSD test for multiple comparisons using R v4.3.2. Principal Component Analysis (PCA) was performed using R v4.0.2. to analyse the variation among the isolates.

## 3 Results

**FIGURE 1:**
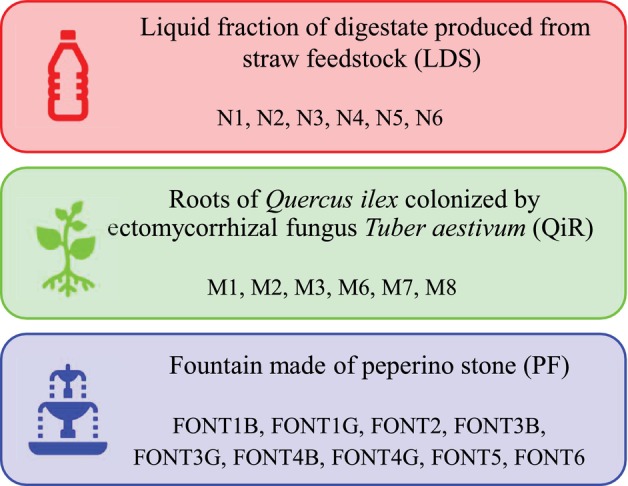
Sources and list of bacterial strains analysed in the study.

A total of 21 morphologically distinct strains were obtained and used for further analysis (six strains from the liquid fraction of straw digestate (LDS), six strains from *Q. ilex* roots (QiR) and nine strains from a fountain made of peperino stone (PF)) (Figure 1).

### 3.1 Molecular Identification

**FIGURE 2:**
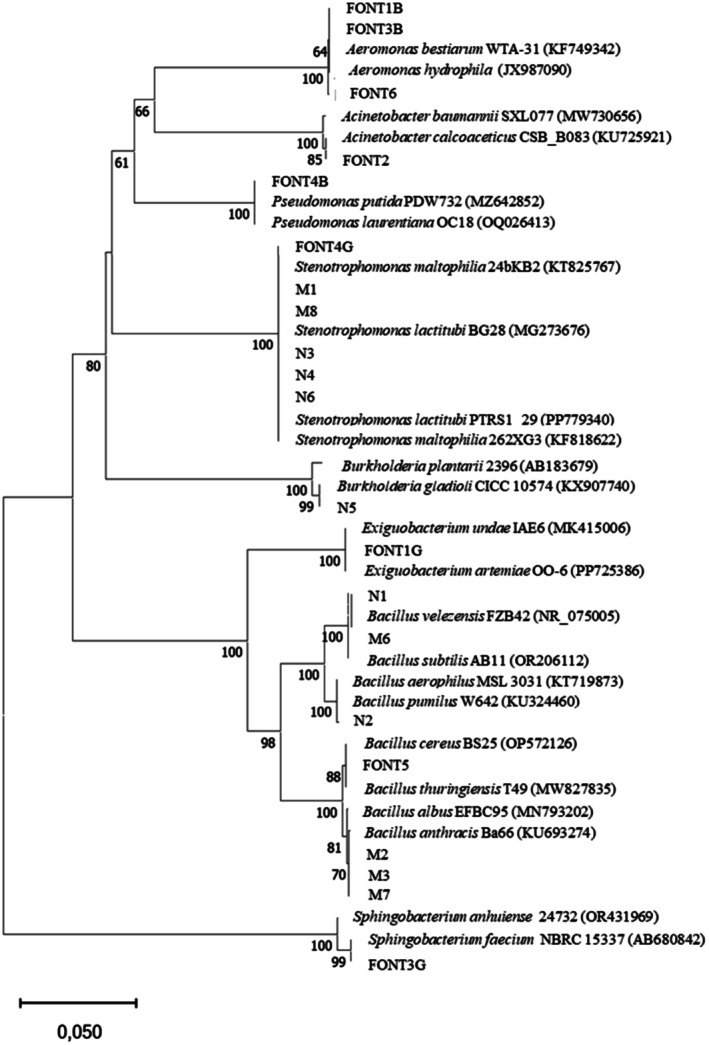
Phylogenetic tree based on the comparison of 16S gene sequences from this study with their closest relatives in GenBank inferred using the Neighbor‐Joining method (the accession numbers are given in parentheses). Bootstrap values ≥ 50% (1000 replicates) are shown below the branches.

The phylogenetic analysis of the 16S rRNA gene indicated that the bacterial isolates were affiliated with eight genera. Among the 21 strains, 7 belonged to the genus *Bacillus*, 6 to the genus *Stenotrophomonas*, and 3 to the genus *Aeromonas*. The genera *Acinetobacter*, *Burkholderia, Exiguobacterium, Pseudomonas* and *Sphingobacterium* were each represented by a single isolate (Figure 2).

### 3.2 Plant Growth Promoting and Metabolite‐Related Traits

**FIGURE 3:**
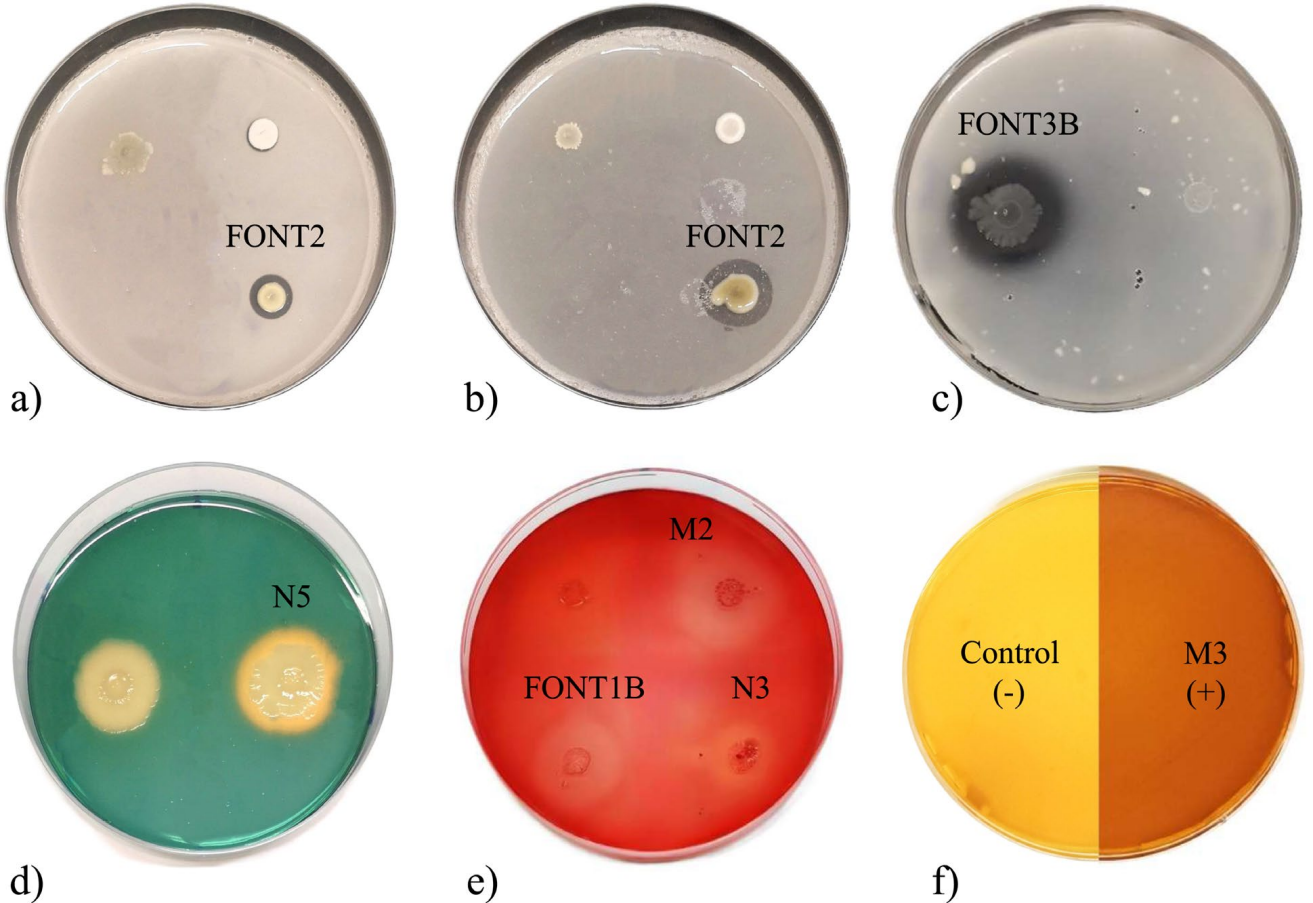
PGP and metabolite‐related traits: (a) Phosphate solubilisation by *Acinetobacter* sp. FONT2; (b) Potassium solubilisation of *Acinetobacter* sp. FONT2; (c) Chitinolytic activity of *Aeromonas* sp. FONT3B; (d) Siderophore production of *Burkholderia* sp. N5; (e) Cellulolytic activity of *Bacillus* sp. M2, *Aeromonas* sp. FONT1B and *Stenotrophomonas* sp. N3; (f) HCN production: Control (−) and *Bacillus* sp. M3 (+).

Most strains exhibited plant growth‐promoting (PGP) and metabolite‐related traits (Figure 3). Among the 21 isolates, 9 (43%) were capable of solubilising phosphorus, while 8 (38%) exhibited potassium‐solubilising activity (Table 1). *Acinetobacter* sp. FONT2 had the highest phosphorus solubilisation index (3.16), followed by *Aeromonas* sp. FONT6 (PSI: 2.98) and *Pseudomonas* sp. FONT4B (PSI: 2.73). The most effective potassium solubilisers included *Acinetobacter* sp. FONT2, *Bacillus* sp. FONT5 and *Aeromonas* sp. FONT6, with potassium solubilisation index (KSI) values of 3.50, 3.10 and 2.98, respectively.

**TABLE 1:** Different plant growth‐promoting traits of bacterial strains isolated from different sources.

Isolates	Sources	PSI	KSI	IAA (48 h) (μg/mL)	IAA (120 h) (μg/mL)
*Bacillus* sp. N1	LDS	—	—	4.46 ± 0.56^h^	3.87 ± 0.13^g^
*Bacillus* sp. N2	LDS	—	—	—	3.61 ± 0.3^g^
*Stenotrophomonas* sp. N3	LDS	—	—	—	—
*Stenotrophomonas* sp. N4	LDS	—	—	—	—
*Burkholderia gladioli* N5	LDS	2.34 ± 0.01^fg^	—	9.37 ± 0.23^g^	8.21 ± 0.36^e^
*Stenotrophomonas* sp. N6	LDS	—	—	—	—
*Stenotrophomonas* sp. M1	QiR	—	—	—	—
*Bacillus* sp. M2	QiR	—	—	2.84 ± 0.28^j^	3.48 ± 0.19^g^
*Bacillus* sp. M3	QiR	—	2.18 ± 0.06^c^	4.91 ± 0.17^h^	8.47 ± 0.19^e^
*Bacillus* sp. M6	QiR	—	—	—	2.71 ± 0.3^h^
*Bacillus* sp. M7	QiR	—	—	—	—
*Stenotrophomonas* sp. M8	QiR	—	—	—	—
*Aeromonas* sp. FONT1B	PF	2.25 ± 0.08^h^	2.23 ± 0.03^c^	32.86 ± 0.19^a^	32.54 ± 0.39^a^
*Exiguobacterium* sp. FONT1G	PF	—	—	—	5.94 ± 0.28^f^
*Acinetobacter* sp. FONT2	PF	3.16 ± 0.06^a^	3.5 ± 0.09^a^	11.51 ± 0.13^f^	—
*Aeromonas* sp. FONT3B	PF	2.41 ± 0.01^f^	2.92 ± 0.15^b^	3.68 ± 0.17^i^	2.32 ± 0.17^h^
*Sphingobacterium* sp. FONT3G	PF	2.63 ± 0.07^d^	—	12.22 ± 0.11^e^	21.86 ± 0.34^c^
*Pseudomonas* sp. FONT4B	PF	2.73 ± 0.01^c^	2.79 ± 0.04^b^	13.32 ± 0.36^d^	20.18 ± 0.13^d^
*Stenotrophomonas* sp. FONT4G	PF	2.50 ± 0.04^e^	2.28 ± 0.06^c^	21.73 ± 0.23^c^	26.00 ± 0.23^b^
*Bacillus* sp. FONT5	PF	2.32 ± 0.04^gh^	3.1 ± 0.05^b^	13.06 ± 0.26^d^	3.55 ± 0.17^g^
*Aeromonas* sp. FONT6	PF	2.98 ± 0.03^b^	2.98 ± 0.1^b^	23.99 ± 0.34^b^	19.99 ± 0.28^d^

*Note:* The values sharing the same lowercase letter within the column are not significantly different (ANOVA; *p* ≤ 0.05, Tukey's HSD test). All values given in the column are the average of three replicates and each experiment was independently repeated twice.

Abbreviations: KSI: potassium solubilisation index; LDS: liquid fraction of digestate produced from straw feedstock; PF: fountain made of peperino stone; PSI: phosphate solubilisation index; QiR: roots of *Q. ilex* colonised by ectomycorrhizal fungus *T. aestivum* .

Twelve bacterial isolates (57%) were capable of producing IAA out of the 21 isolates tested after 48 h. *Bacillus* sp. N2 and M6, as well as *Exiguobacterium* sp. FONT1G, produced IAA after 120 h of incubation. The isolates that produced the highest levels of IAA were *Aeromonas* sp. FONT1B, *Stenotrophomonas* sp. FONT4G and *Aeromonas* sp. FONT6, with values exceeding 21.73 μg/mL (Table 1).

Most of the strains (11 out of 21; 52%), primarily belonging to the genus *Bacillus*, demonstrated siderophore production by altering the colour of the CAS media from blue to yellow.

Screened bacterial isolates were evaluated for indirect PGP traits based on their production of extracellular hydrolytic enzymes (Table 2). A total of 13 isolates (62%) produced cellulase, 15 strains produced chitinase, while 4 isolates exhibited no enzymatic activities.

**TABLE 2:** Different metabolite‐related traits of bacterial strains isolated from different sources.

Isolates	Sources	CHI	CI	SPI	HCN
*Bacillus* sp. N1	LDS	−	2.43 ± 0.04^def^	−	−
*Bacillus* sp. N2	LDS	−	−	2.39 ± 0.07^bc^	−
*Stenotrophomonas* sp. N3	LDS	2.67 ± 0.08^c^	3.19 ± 0.14^c^	−	−
*Stenotrophomonas* sp. N4	LDS	2.5 ± 0.28^cde^	−	−	+
*Burkholderia gladioli* N5	LDS	2.36 ± 0.25^def^	3.9 ± 0.14^b^	2.53 ± 0.05^b^	−
*Stenotrophomonas* sp. N6	LDS	2.71 ± 0.07^bc^	2.73 ± 0.1^d^	−	−
*Stenotrophomonas* sp. M1	QiR	2.64 ± 0.05^cd^	−	2.28 ± 0.04^cde^	−
*Bacillus* sp. M2	QiR	2.63 ± 0.07^cd^	4 ± 0.23^b^	2.21 ± 0.08^de^	+
*Bacillus* sp. M3	QiR	2.57 ± 0.01^cde^	5.72 ± 0.27^a^	2.47 ± 0.11^b^	+
*Bacillus* sp. M6	QiR	−	−	2.32 ± 0.06^cd^	−
*Bacillus* sp. M7	QiR	2.35 ± 0.09^def^	−	2.18 ± 0.1^de^	−
*Stenotrophomonas* sp. M8	QiR	2.44 ± 0.05^cde^	2.62 ± 0.17^de^	−	−
*Aeromonas* sp. FONT1B	PF	2.32 ± 0.03^ef^	3.47 ± 0.15^c^	2.73 ± 0.09^a^	+
*Exiguobacterium* sp. FONT1G	PF	2.37 ± 0.15^def^	2.54 ± 0.12^def^	2.3 ± 0.06^cde^	−
*Acinetobacter* sp. FONT2	PF	−	−	2.3 ± 0.05^cde^	+
*Aeromonas* sp. FONT3B	PF	2.59 ± 0.06^cde^	2.4 ± 0.07^ef^	−	+
*Sphingobacterium* sp. FONT3G	PF	−	2.29 ± 0.18^f^	−	−
*Pseudomonas* sp. FONT4B	PF	−	−	−	−
*Stenotrophomonas* sp. FONT4G	PF	4.06 ± 0.31^a^	−	2.16 ± 0.09^e^	−
*Bacillus* sp. FONT5	PF	2.13 ± 0.09^f^	2.62 ± 0.16^de^	−	+
*Aeromonas* sp. FONT6	PF	3 ± 0.13^b^	3.85 ± 0.06^b^	−	−

*Note:* The values sharing the same lowercase letter within the column are not significantly different (ANOVA; *p* ≤ 0.05, Tukey's HSD test). All values given in the column are the average of three replicates, and each experiment was independently repeated twice.

Abbreviations: CHI: chitinolytic activity index; CI: cellulolytic activity index; LDS: liquid fraction of digestate produced from straw feedstock; PF: fountain made of peperino stone; QiR: roots of *Q. ilex* colonised by ectomycorrhizal fungus *T. aestivum* ; SPI: siderophore production index.

Production of hydrogen cyanide (HCN) was observed in 7 bacterial strains (33%): *Stenotrophomonas* sp. N4, *Bacillus* sp. M2 and M3, *Aeromonas* sp. FONT1B, *Acinetobacter* sp. FONT2, *Aeromonas* sp. FONT3B and *Bacillus* sp. FONT5 (Table 2).

**FIGURE 4:**
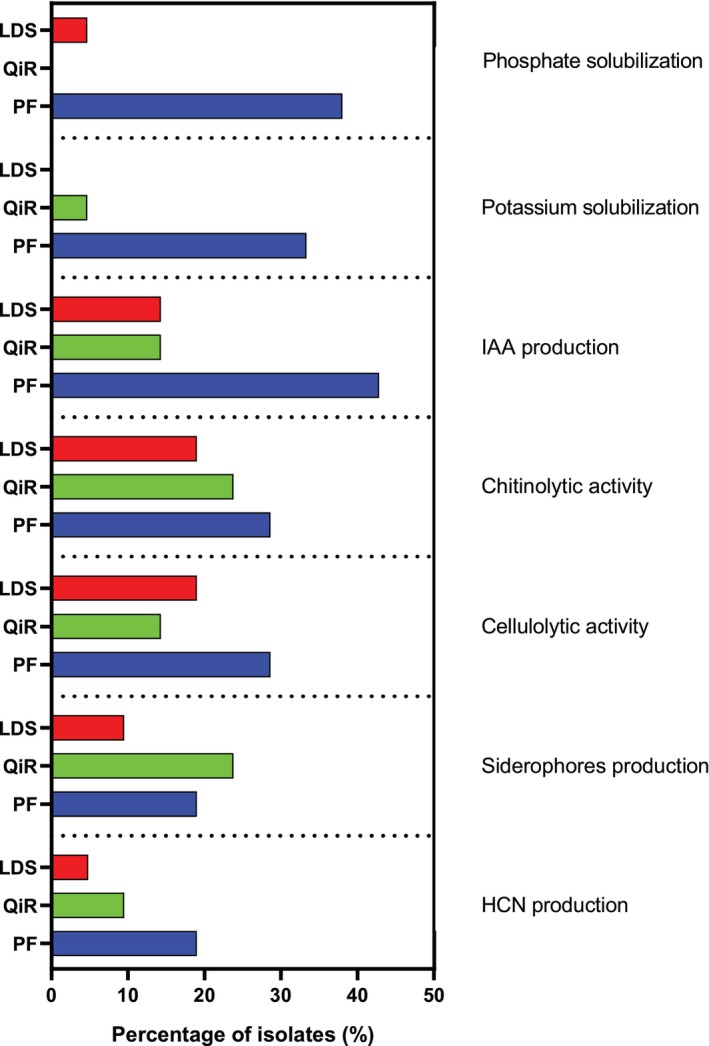
Percentage of isolates (%) exhibiting PGP and metabolite‐related traits, grouped according to their isolation source. LDS: Liquid fraction of digestate produced from straw feedstock; PF: Fountain made of peperino stone; QiR: Roots of *Q. ilex* colonised by ectomycorrhizal fungus *T. aestivum* .

It is worth noting that bacteria obtained from the PF exhibited more PGP and metabolite‐related traits than those sourced from other locations (Figure 4). Specifically, 66.6% of the taxa isolated from PF demonstrated at least 5 PGP properties and metabolite‐related traits, followed by 16.7% and 33.3% of the bacteria acquired from the LDS and QiR, respectively. All 21 bacteria isolated showed at least two PGP properties (Table 1). The production of IAA, HCN and siderophores, along with chitinolytic and cellulolytic activities, was the most prevalent characteristic among the isolated bacteria. The ability to solubilise phosphate and potassium was not found in bacteria isolated from QiR and LDS, respectively.

### 3.3 Abiotic Stress Tolerance

Table 3 summarises the abiotic stress tolerance traits exhibited by the 21 bacterial strains.

**TABLE 3:** Abiotic stresses tolerance of bacterial strains obtained from distinct sources (LDS, QiR and PF).

Isolates	Sources	Salinity tolerance (%)				pH tolerance					Drought stress, osmotic stress (drought) tolerance (MPa)		
		5.0	7.5	10.0	15.0	4	5	6	8	10	−0.35	−0.75	−1.2
*Bacillus* sp. N1	LDS												
*Bacillus* sp. N2	LDS												
*Stenotrophomonas* sp. N3	LDS												
*Stenotrophomonas* sp. N4	LDS												
*Burkholderia gladioli* N5	LDS												
*Stenotrophomonas* sp. N6	LDS												
*Stenotrophomonas* sp. M1	QiR												
*Bacillus* sp. M2	QiR												
*Bacillus* sp. M3	QiR												
*Bacillus* sp. M6	QiR												
*Bacillus* sp. M7	QiR												
*Stenotrophomonas* sp. M8	QiR												
*Aeromonas* sp. FONT1B	PF												
*Exiguobacterium* sp. FONT1G	PF												
*Acinetobacter* sp. FONT2	PF												
*Aeromonas* sp. FONT3B	PF												
*Sphingobacterium* sp. FONT3G	PF												
*Pseudomonas* sp. FONT4B	PF												
*Stenotrophomonas* sp. FONT4G	PF												
*Bacillus* sp. FONT5	PF												
*Aeromonas* sp. FONT6	PF												

*Note:* Key: Colours: positive; White: negative.

Abbreviations: LDS: liquid fraction of digestate produced from straw feedstock; PF: fountain made of peperino stone; QiR: roots of *Q. ilex* colonised by ectomycorrhizal fungus *T. aestivum* .

A total of 13 isolates exhibited salt tolerance; 4 strains demonstrated the ability to grow only in the presence of 5% NaCl. Nine isolates could tolerate salt concentrations up to 7.5%, while only two strains (*Bacillus* sp. M7 and *Stenotrophomonas* sp. M8) could grow at a 15% concentration. All strains grew within a pH range of 5 to 8, while only some could tolerate more extreme conditions, with 14 isolates surviving at pH 4 and 17 at pH 10. All strains isolated from the PF showed resistance to osmotic stress at the lowest dose (−0.35 MPa), with *Acinetobacter* sp. FONT2 being the most tolerant to osmotic stress at −1.2 MPa.

### 3.4 Seed Germination Test

No significant differences were observed between the controls and the seeds treated with the different bacterial isolates (ANOVA, *p* > 0.05) (Table S1).

### 3.5 Antagonistic Activity Against *Phytophthora infestans*

**FIGURE 5:**
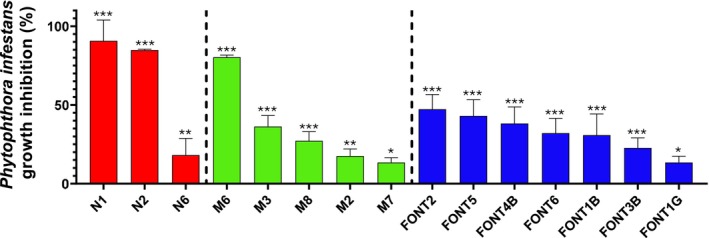
Percentage of growth inhibition of *P. infestans* AMV23 co‐cultured with the bacterial strains. The asterisks indicate significant differences between control and bacterial treatments. ANOVA: *** (*p* ≤ 0.001), ** (*p* ≤ 0.01) and * (*p* ≤ 0.05).

Most of the strains (71%) significantly reduced the growth of *P. infestans* (Figure 5) (ANOVA, *p* < 0.05). The most effective was *Bacillus* sp. N1, which inhibited pathogen growth by 91%, followed by *Bacillus* sp. N2 and M6, with colony growth reduction rates of 85% and 80%, respectively (Figure 5).

### 3.6 Variation Among Bacteria From Different Sources

**FIGURE 6:**
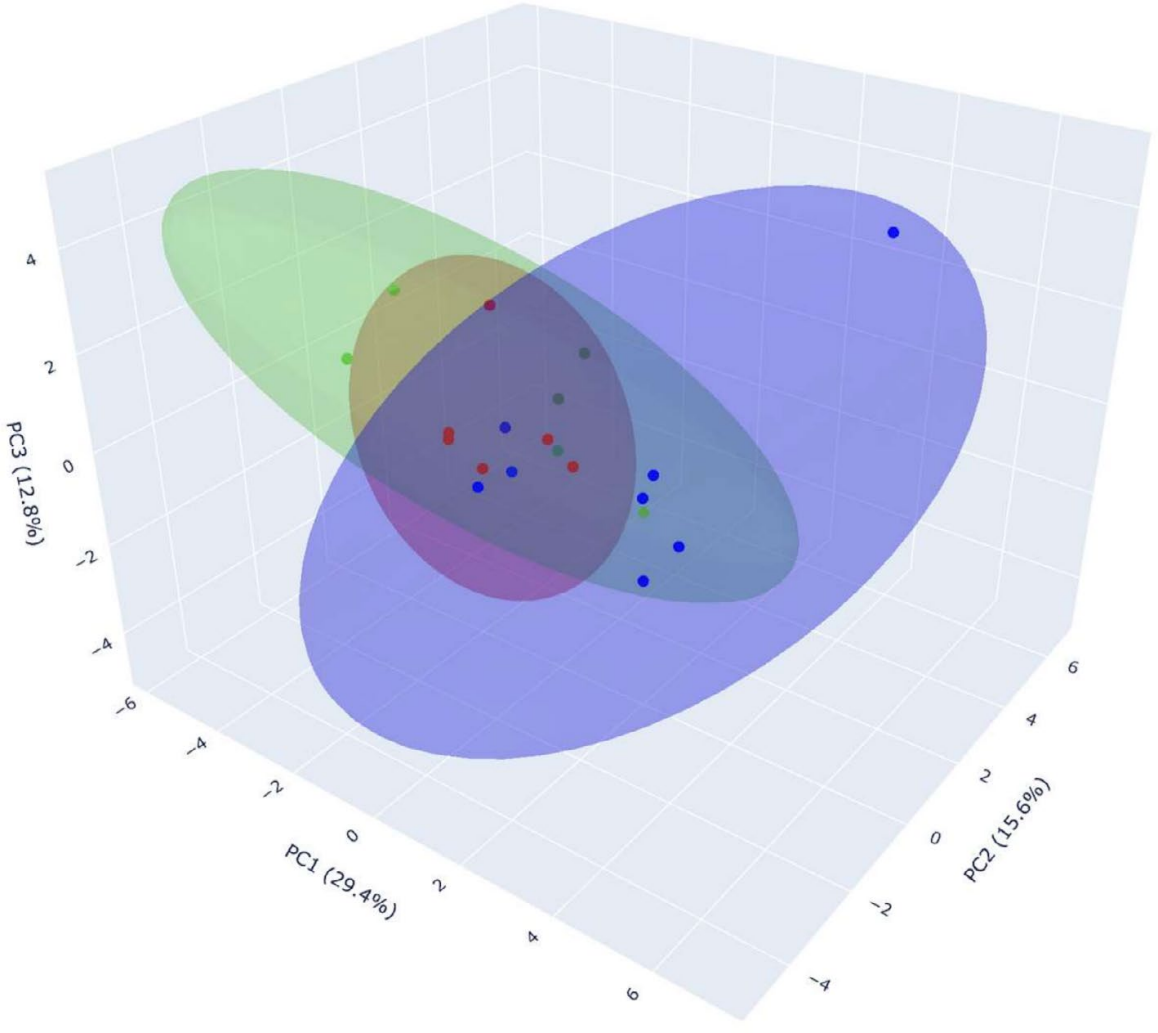
Three‐dimensional PCA of the 21 bacterial isolates, performed on PGP traits, mycolytic enzyme production and response to abiotic and biotic stress conditions. Each point represents an isolate, coloured according to its group: LDS (red), QiR (bright green), PF (blue). LDS: Liquid fraction of digestate produced from straw feedstock; PF: Fountain made of peperino stone; QiR: Roots of *Q. ilex* colonised by ectomycorrhizal fungus *T. aestivum* . The semi‐transparent ellipsoids visualise the 95% confidence regions for each group.

Principal Component Analysis was carried out to highlight the variation in bacterial PGP, metabolite‐related traits and tolerance to biotic and abiotic stress among sources (Figure 6). The first three principal components (PC1, PC2, PC3) capture most of the total variance present in the dataset, accounting for 29.4%, 12.8% and 15.6% of the variation, respectively. Group LDS is compact and well defined, indicating low internal variability, while Group QiR is more dispersed, showing functional heterogeneity. Group PF appears distinct, likely associated with traits such as IAA production or pH response. Table 4 reports the loading values associated with the first three principal components of the PCA. Saline conditions and acidic pH are the main drivers in the multivariate distribution of the samples. PC1 captures salt tolerance and solubilisation traits. PC2 mostly reflects pH response and enzymatic activity. PC3 is strongly tied to high salt and drought stress (Figure 6). An interactive 3D PCA plot with confidence ellipsoids is provided as Supporting Information to facilitate further data exploration (Figure S1).

**TABLE 4:** PCA loadings and covariable contributions.

Variable		PC1	PC2	PC3	PC1 (%)	PC2 (%)	PC3 (%)
Salinity tolerance (%)	**5.0**	−0.381	−0.036	0.097	**14.49**	0.13	0.94
Salinity tolerance (%)	**7.5**	−0.254	−0.266	0.219	6.46	7.05	4.79
Salinity tolerance (%)	**10.0**	−0.2	−0.279	0.351	4.01	7.78	**12.33**
Salinity tolerance (%)	**15.0**	−0.193	−0.192	0.311	3.74	3.7	9.66
pH tolerance	**4**	0.105	−0.388	0.211	1.1	**15.08**	4.47
pH tolerance	**10**	0.147	−0.386	0.186	2.15	**14.91**	3.46
Osmotic stress (drought) tolerance (MPa)	**−0.35**	0.338	−0.175	0.032	**11.44**	3.07	0.1
Osmotic stress (drought) tolerance (MPa)	**−0.75**	0.23	0.223	0.456	5.27	4.99	**20.8**
Osmotic stress (drought) tolerance (MPa)	**−1.2**	0.23	0.223	0.456	5.27	4.99	**20.8**
PSI		0.345	−0.246	0.071	**11.87**	6.04	0.51
KSI		0.392	−0.089	−0.033	**15.38**	0.8	0.11
IAA production (48 h) (μg/mL)		0.327	−0.118	−0.099	**10.69**	1.4	0.98
CHI		−0.056	−0.233	−0.27	0.32	5.42	7.29
CI		−0.008	−0.318	−0.289	0.01	**10.13**	8.37
SPI		−0.038	0.357	0.111	0.15	**12.75**	1.24
HCN production		0.273	0.127	−0.164	7.46	1.62	2.69
Biocontrol		0.046	0.036	0.121	0.21	0.13	1.45

*Note:* Dominant contributors are reported in bold.

Abbreviations: CHI: chitinolytic activity index; CI: cellulolytic activity index; KSI: potassium solubilisation index; PSI: phosphate solubilisation index; SPI: siderophores production index.

## 4 Discussion

Environmental conditions, encompassing various elements such as climate change, pollution and habitat disruption, present significant threats to agriculture, urban areas and forest ecosystems (Mehta 2024; Mishra 2025). These factors can inhibit the growth of numerous plant species, reduce overall biodiversity and increase vulnerability to pests and diseases (Austin and Ballaré 2023; Kumar, Rithesh, et al. 2023; Kumar, Thilagam, et al. 2023; Kaplan et al. 2024). Therefore, harnessing the dual capacity of certain beneficial bacteria, serving as both growth promoters and stress‐mitigating agents for plants, can boost crop productivity and enhance long‐term agricultural sustainability (Hakim et al. 2021; Fadiji et al. 2022; Abou Jaoudé et al. 2024; Ansabayeva et al. 2025).

In this study, 21 bacterial strains were isolated from three distinct sources: straw digestate (LDS), *Q. ilex* roots mycorrhised by *T. aestivum* (QiR), and a peperino stone fountain (PF). These bacterial isolates were thoroughly characterised for their potential to promote plant growth and control *P. infestans* the causal agent of potato and tomato late blight (Nowicki et al. 2012).

Furthermore, their tolerance to stress conditions, including salinity, pH variations and osmotic stress, was also evaluated. The taxa were classified into eight genera: *Acinetobacter, Aeromonas, Bacillus*, *Burkholderia, Exiguobacterium*, *Pseudomonas*, *Sphingobacterium* and *Stenotrophomonas*. The genera identified in this study are well known for their pivotal and influential roles as PGP bacteria. In addition, they act not only as enhancers of plant growth but also as agents that improve stress resilience across various plant species. For instance, numerous strains within the genus *Bacillus* have been reported to improve plant tolerance to abiotic stresses, including drought, salinity and heavy metals. This is achieved through the induction of systemic tolerance, the modulation of antioxidant systems and the enhancement of water and nutrient uptake (Choudhary and Johri 2009; Dimkpa et al. 2009; Idris et al. 2007; Vardharajula et al. 2011). Additionally, many *Bacillus* strains are widely recognised for their ability to control various phytopathogens, including *Alternaria* spp., *Fusarium solani*, *P. infestans* and *Verticillium dahliae* (Dhouib et al. 2019; Lim et al. 2017; Yan et al. 2021). Similarly, *Acinetobacter* and *Aeromonas* have been associated with improved plant nutrient uptake. Additionally, these microorganisms increase plant tolerance to drought and salinity stress, enabling plants to survive and thrive under adverse environmental conditions. They also enhance plant disease tolerance by producing antimicrobial compounds. Bacteria can also synthesise siderophores, which chelate and transport iron, a critical nutrient that promotes plant health. By helping plants withstand various stress factors, these microorganisms contribute to overall plant well‐being and resilience (Marfetán et al. 2023; Mehnaz et al. 2001; Papade et al. 2024; Suzuki et al. 2014; Trotel‐Aziz et al. 2008). The genus *Exiguobacterium* has been shown to significantly enhance the growth of various important plant species (Kasana and Pandey 2018). Notably, the strain *Exiguobacterium* sp. S17 has been shown to promote the growth of *Brassica juncea* (Indian mustard), *Beta vulgaris* (chard) and *Lactuca sativa* (lettuce) plants by more than 100%, compared to control plants. Similarly, *Exiguobacterium* NII‐0906 has demonstrated a significant ability to promote both root and shoot lengths of *Vigna unguiculata* (cowpea) seedlings and to inhibit in vitro the growth of common phytopathogenic fungi, such as *Penicillium expansum*, *Geotrichum candidum*, *F. oxysporum* and *Aspergillus niger* by 78%, 87%, 69% and 89%, respectively (Dastager et al. 2010). The genus *Burkholderia* comprises a group of bacteria known for their versatile roles across various ecological niches. Notably, these bacteria can enhance plant growth and have gained increasing importance due to their ability to inhibit pathogenic microorganisms both directly and through the production of antagonistic metabolites (Eberl and Vandamme 2016; Elshafie and Camele 2021). The unique capabilities of these bacteria lie in their diverse production of bioactive compounds, including siderophores, enzymes like proteases, chitinases, amylases and cellulases, secondary metabolites and VOCs, which play significant roles in microbial antagonism and plant enhancement (Vial et al. 2007; Chen et al. 2020; Barrera‐Galicia et al. 2021). Some species have been confirmed to promote plant growth and enhance plant resistance to biotic and abiotic stresses, promising avenues for application (Suárez‐Moreno et al. 2012). Wang et al. (2024) reported that *B. gladioli* KRS027 enhanced the growth and development of the stem, shoot apical meristem and root apical tissue division in cotton seedlings by modulating the biosynthesis and signalling pathways of brassinosteroid, gibberellins and auxins. *Burkholderia gladioli* KRS027 also exhibited broad‐spectrum antifungal activity, effectively suppressing grey mould disease caused by *Botrytis cinerea*, and stimulates plant immunity via ISR activated by salicylic acid, jasmonic acid and ethylene‐dependent signalling pathways (Wang et al. 2023). *Pseudomonas* strains exhibit strong survival under stressful environmental conditions and are particularly effective in suppressing diseases like root rot, caused by plant pathogens including *Phytophthora* spp., *F. solani* and *Rhizoctonia solani* (Kong et al. 2023; Sharma et al. 2022; Song et al. 2022). Furthermore, these bacteria play a crucial role in promoting plant growth and provide numerous benefits to agricultural systems (Dorjey et al. 2017). Several members of the genus *Sphingobacterium* have been characterised for their PGP traits (Ali et al. 2015; Hagaggi and Abdul‐Raouf 2022; Vaishnav et al. 2020). Some isolates, such as *Sphingobacterium thalpophilum* NMS02 S296, an endophyte isolated from the bracts of *Musa* spp. (banana), possess notable antagonistic properties against *Fusarium* and are considered a natural and sustainable solution for managing banana wilt (Ajesh et al. 2024). Certain *Stenotrophomonas* species have gained attention for their ability to improve plant growth under saline and drought conditions (Kumar, Rithesh, et al. 2023; Kumar, Thilagam, et al. 2023; Sharma et al. 2023; Zhao et al. 2024). This is achieved through the production of ACC deaminase and antioxidant enzymes. Moreover, these species possess the ability to solubilise phosphate, thereby enhancing nutrient accessibility, as well as to suppress soil‐borne pathogens such as *R. solani* and *F. oxysporum* (Ryan et al. 2009; Singh and Jha 2017; Ulrich et al. 2021).

Our study has expanded our understanding of the ecological niches occupied by PGP bacteria. All newly isolated strains possess traits that can aid in plant growth promotion, disease control and stress tolerance. As shown in Table 1, 38% (8 out of 21) of the strains produced indole compounds and were capable of solubilising phosphorus and/or potassium, all of which contribute to plant growth under stress conditions (Gupta et al. 2021; Timofeeva et al. 2023). Phosphorus and potassium solubilisation is essential for key physiological processes such as enzyme activation and osmoregulation, particularly during drought stress (Khan et al. 2023). Additionally, indole‐3‐acetic acid (IAA) promotes root formation and elongation, boosting the plant's ability to access water and nutrients, and thereby increasing resilience to both abiotic and biotic stresses (Hyder et al. 2020; Lebrazi et al. 2020; Phyu et al. 2019). This aligns with Shahab et al. (2009), who reported that most phosphate‐solubilising bacteria produce IAA. Despite variations in their PSI and KSI indices (Table 1), 7 of the 21 tested strains (33.3%) solubilised both phosphorus and potassium salts. The isolates that produced the highest levels of IAA were the *Aeromonas* FONT1B and FONT6 strains (Table 1). Over half of the isolates (52%) scavenged iron from the environment by producing siderophores (Table 2). Iron uptake plays a crucial role in helping plants cope with abiotic stresses such as drought, heavy metal detoxification and salt stress, while also inhibiting plant pathogens (Gu et al. 2020). More than half of the isolates from each group produced chitinases and cellulases (Table 2). These enzymes enhance plant resilience to pests and pathogens by hydrolysing the structural components of their cell walls (Kavino et al. 2010). Data reported in Table 2 also indicated that approximately 81% of the isolates produced lytic enzymes, which were sometimes associated with the production of the antifungal metabolite hydrogen cyanide (HCN). While most bacterial strains tolerated a wide pH spectrum (pH 4–10), only a limited number of taxa exhibited growth at salt concentrations exceeding 5% and under osmotic conditions below −0.35 MPa.

A significant finding of this study was that most of the isolates exhibited biocontrol activity against *P. infestans* , a major oomycete pathogen responsible for significant crop losses (Figure 5). Interestingly, the *Bacillus* strains N1 and N2 from LDS and M6 from QiR exhibited the highest inhibition percentage against the pathogen. The effectiveness of the other *Bacillus* strains isolated from QiR and PF in inhibiting *P. infestans* was less pronounced (Figure 5), which may reflect genetic variability due to the taxon‐environment relationship (Saito et al. 2020). Similar results were observed for the four *Stenotrophomonas* strains, which were equally distributed in two groups: no inhibition and low inhibition (less than 30%; Figure 5).

Our data indicated that no direct correlation between the production of cellulolytic activities and the biocontrol of *P. infestans* occurred. Strains *Bacillus* sp. N2 and M6, and *Acinetobacter* sp. FONT2, which were among the best *P. infestans* biocontrol agents isolated from the three environments, did not produce any cellulase (Table 2). Cellulases degrade cellulose, typically accounting for approximately 32%–35% of the *Phytophthora* spp. cell wall mass (Pang et al. 2020). Interestingly, in addition to their anti‐oomycete activity, the isolates FONT1B, FONT3B, FONT5 and M3 displayed at least five distinct traits (Tables 1 and 2), which are associated with promoting plant growth and enhancing plant stress resilience (Kim et al. 2000; Scannell et al. 1972; Wanga et al. 1999). Another notable finding of our study was the distinct functional patterns observed among microbial groups obtained from different sources, which could guide strain selection for the production of biofertilisers, biocontrol application and ecological studies.

As reported in Table 3, the isolates from LDS and QiR exhibit higher tolerance to saline environments. In contrast, isolates from PF demonstrated tolerance to osmotic stress up to at least −0.35 MPa (Table 3). This trait is valuable for survival under harsh conditions, such as drought stress. Unexpectedly, as observed for *Acinetobacter* sp. strain FONT2, the high osmotic stress tolerance was not associated with saline tolerance (Table 3). *Bacillus* and *Stenotrophomonas* strains exhibiting saline tolerance up to 15% were observed among isolates from QiR only (Table 3). Interestingly, both strains (M7 and M8) also displayed a broad pH tolerance, growing at pH values ranging from 4 to 10. Except for *Bacillus* sp. M3, none of the *Bacillus* and *Stenotrophomonas* strains from LDS and QiR solubilised phosphorus or potassium. In contrast, the *Bacillus* and *Stenotrophomonas* strains isolated from PF solubilised both elements (Table 1). Interestingly, regardless of taxon, none of the strains from QiR solubilised phosphate, and none of the isolates from LDS solubilised potassium (Figure 3). *Burkholderia gladioli* N5 was the only isolate from LDS that solubilised phosphate and exhibited the highest number of plant‐beneficial traits (5 out of 7) among the strains from LDS (Tables 1 and 2), consistent with the metabolic versatility characteristic of members of the *Burkholderia* genus. Except for *Stenotrophomonas* sp. N4, none of the *Stenotrophomonas* strains produce HCN (Table 2). This trait was observed in 2 of the 7 *Bacillus* strains (one of which was isolated from PF) and 2 of the 3 *Aeromonas* strains (both isolated from PF), as well as in *Acinetobacter* sp. FONT2 (Table 2). The functional versatility of the members of the PF group, which exhibits the highest relative percentage of isolates with PGP and metabolite‐related traits (except for siderophore production) (Figure 3), likely reflects the adaptation of the PF‐associated bacteria to the challenging conditions of stone surfaces. These environments are characterised by temperature fluctuations, limited nutrient availability and intense microbial competition. Such conditions may act as selective pressures, promoting the development of bacteria with enhanced survival strategies and beneficial traits.

## 5 Conclusions

In conclusion, the approach used in this study enabled the targeted selection of microbial sources, focusing on environmental niches or plant waste, which are more likely to harbour beneficial traits and stress‐related properties. Among the isolates tested, the FONT1B strain showed the most promising performance in terms of biocontrol potential. The bacterial strains identified, particularly FONT1B, represent strong candidates for the development of eco‐friendly, cost‐effective and user‐friendly biofertilisers or biopesticides, with potential applications in managing *Phytophthora*‐related diseases and improving crop productivity under climate‐induced stress conditions. While these results are promising, they do not fully represent the complexity of real biological and environmental interactions. Therefore, further in vivo studies are essential to validate the effectiveness, safety and practical applicability of biocontrol agents under natural conditions.

## Author Contributions

**Chiara Antonelli:** methodology, investigation. **Michele Narduzzi:** methodology, investigation. **Maurizio Ruzzi:** writing – review and editing. **Antonino Testa:** writing – review and editing. **Anna Maria Vettraino:** writing – review and editing, writing – original draft, visualisation, validation, supervision, project administration, methodology, investigation, funding acquisition, formal analysis, conceptualization.

## Conflicts of Interest

The authors declare no conflicts of interest.

## Supporting information


**Figure S1:** 3D PCA plot: An interactive 3D PCA plot with confidence ellipsoids of the 21 bacterial isolates, performed on PGP traits, mycolytic enzyme production and response to abiotic and biotic stress conditions. Each point represents an isolate, coloured according to its group: LDS (red), QiR (bright green), PF (blue). LDS: Liquid fraction of digestate produced from straw feedstock; QiR: Roots of *Q. ilex* colonised by ectomycorrhizal fungus *T. aestivum* ; PF: Fountain made of peperino stone. The semi‐transparent ellipsoids visualise the 95% confidence regions for each group.

**Table S1:** Effect of selected bacteria isolates on tomato seed germination.

## Data Availability

The data that support the findings of this study are available on request from the corresponding author. The data are not publicly available due to privacy or ethical restrictions.
