# Supplementary material for: Mining Microbial Niches: Sources of Bacteria for Enhancing Plant Growth and Resilience to pH, Salinity, Drought and Phytophthora infestans
Source: Environ Microbiol Rep. 2025 Oct 18;17(5):e70217. doi: 10.1111/1758-2229.70217 (PMC12535207; doi:10.1111/1758-2229.70217)
Supplement: Supplementary file 2 — Table S1: Effect of selected bacteria isolates on tomato seed germination. [file EMI4-17-e70217-s002.docx]

**Table S1.** Effect of selected bacteria isolates on tomato seed germination

| **Isolate** | **Seed germination**  **(%)** |
| --- | --- |
| Control | 90.00 ^a^ |
| *Bacillus* sp. **N1** | 87.78 ^a^ |
| *Bacillus* sp. **N2** | 87.78 ^a^ |
| *Stenotrophomonas* sp. **N3** | 87.78 ^a^ |
| *Stenotrophomonas* sp. **N4** | 90.00 ^a^ |
| *Burkholderia gladioli* **N5** | 90.00 ^a^ |
| *Stenotrophomonas* sp. **N6** | 88.89 ^a^ |
| *Stenotrophomonas* sp. **M1** | 94.44 ^a^ |
| *Bacillus* sp. **M2** | 92.22 ^a^ |
| *Bacillus* sp. **M3** | 91.11 ^a^ |
| *Bacillus* sp. **M6** | 92.22 ^a^ |
| *Bacillus* sp. **M7** | 85.56 ^a^ |
| *Stenotrophomonas* sp. **M8** | 91.11 |
| *Aeromonas* sp. **FONT1B** | 94.44 ^a^ |
| *Exiguobacterium* sp. **FONT1G** | 85.56 ^a^ |
| *Acinetobacter* sp. **FONT2** | 92.22 ^a^ |
| *Aeromonas* sp. **FONT3B** | 94.44 ^a^ |
| *Sphingobacterium* sp. **FONT3G** | 88.89 ^a^ |
| *Pseudomonas* sp. **FONT4B** | 88.89 ^a^ |
| *Stenotrophomonas* sp. **FONT4G** | 91.11 ^a^ |
| *Bacillus* sp. **FONT5** | 91.11 ^a^ |
| *Aeromonas* sp. **FONT6** | 88.89 ^a^ |

All values given in the column are the percentage of three replicates, and the test was independently repeated twice. The values sharing the same lowercase letter within the column are not significantly different (ANOVA; p ≤ 0.05, Tukey’s HSD test).
